# Rapid Microfluidic Preparation of Niosomes for Targeted Drug Delivery

**DOI:** 10.3390/ijms20194696

**Published:** 2019-09-22

**Authors:** Didem Ag Seleci, Viktor Maurer, Frank Stahl, Thomas Scheper, Georg Garnweitner

**Affiliations:** 1 Institute for Particle Technology (iPAT), Technische Universität Braunschweig, 38104 Braunschweig, Germany; v.maurer@tu-braunschweig.de (V.M.); g.garnweitner@tu-braunschweig.de (G.G.); 2 Centre for Pharmaceutical Engineering Research (PVZ), Technische Universität Braunschweig, 38106 Braunschweig, Germany; 3 Institute for Technical Chemistry, Leibniz University Hannover, 30167 Hannover, Germany; stahl@iftc.uni-hannover.de (F.S.); scheper@iftc.uni-hannover.de (T.S.)

**Keywords:** niosomes, microfluidics, targeted drug delivery, glioma

## Abstract

Niosomes are non-ionic surfactant-based vesicles with high promise for drug delivery applications. They can be rapidly prepared via microfluidics, allowing their reproducible production without the need of a subsequent size reduction step, by controlled mixing of two miscible phases of an organic (lipids dissolved in alcohol) and an aqueous solution in a microchannel. The control of niosome properties and the implementation of more complex functions, however, thus far are largely unknown for this method. Here we investigate microfluidics-based manufacturing of topotecan (TPT)-loaded polyethylene glycolated niosomes (PEGNIO). The flow rate ratio of the organic and aqueous phases was varied and optimized. Furthermore, the surface of TPT-loaded PEGNIO was modified with a tumor homing and penetrating peptide (tLyp-1). The designed nanoparticular drug delivery system composed of PEGNIO-TPT-tLyp-1 was fabricated for the first time via microfluidics in this study. The physicochemical properties were determined through dynamic light scattering (DLS) and zeta potential analysis. In vitro studies of the obtained formulations were performed on human glioblastoma (U87) cells. The results clearly indicated that tLyp-1-functionalized TPT-loaded niosomes could significantly improve anti-glioma treatment.

## 1. Introduction

Topotecan (TPT), a water-soluble analog of camptothecin, is a DNA topoisomerase I inhibitor, thereby interfering with DNA replication and DNA repair [1]. It has shown good antitumor activity against a wide variety of solid tumors including breast cancer, ovarian, and cervical cancer as well as glioma [2,3]. The major limitation of this drug is that TPT is unstable in physiological conditions and undergoes a pH-dependent rapid and reversible hydrolysis from a closed lactone ring to the inactive carboxylated open-ring form, which causes the loss of the antitumor activity of the drug [4]. To protect TPT from hydrolysis, the encapsulation into different types of nanoparticles such as liposomes, lipid nanoparticles, and mesoporous silica has been presented [5,6,7,8].

A variety of vesicular nanocarriers have been developed to enhance the stability of drugs and adjust their regulated release while reducing possible side effects. Among the most studied vesicles, non-ionic surfactant-based vesicles, so-called “niosomes”, have gained a lot of attention recently due to their low cost, lower toxicity, and long-term stability compared to other nanoscale vehicles (e.g., liposomes) [9,10,11]. Niosomes consist of non-ionic surfactants, cholesterol, specific additives, and a hydration medium. The self-assembly of non-ionic surfactants in aqueous media results in the formation of closed bi-layered vesicles [12]. The ability to accommodate a variety of drugs with a broad spectrum of solubilities implies a great potential of niosomes for future therapeutic applications [13,14].

Even though the preparation of niosomes has been investigated in a number of previous studies, their synthesis still remains challenging. Numerous different bulk methods have been investigated to prepare niosomes (e.g., thin-film hydration, reversed-phase evaporation, heating methods) that, however, require a post-synthetic size altering procedure (extrusion, sonication) in order to attain homogeneous and unilamellar particles of a specific size [15,16,17]. Since the physicochemical properties and thus the pharmacokinetics of the vesicles depends on the formulation and manufacturing method, the synthesis is a crucial factor for the development of new drug vehicles [18]. Considering the relative complexity of the conventional methods, the development of reproducible and scalable fabrication procedures is of essential importance to enable their implementation in drug delivery applications.

Microfluidic systems have proven to be a great platform for the synthesis and optimization of various nanoparticles including metal and metal oxide nanoparticles, semiconductors, colloidal nanoparticles, and hybrid organic–inorganic nanoparticle composites [19,20,21,22,23]. The formation of niosomes can be achieved via microfluidics by applying a controlled laminar mixing of two or more inlet streams, whereupon precipitation of the surfactant and subsequent self-assembly into the product occur [24,25]. By precisely adjusting the flow rates of the aqueous and organic streams, defined vesicles of a specific size can be obtained while lowering the required liquid volumes and thereby reducing the preparation time and development costs compared to other methods [26,27]. Despite extensive research concerning different microfluidics parameters, the effect of the encapsulated drug on the niosome properties has not been fully explored yet. Further investigations are required to gain an understanding of the influence of different formulations and flow rates to achieve the microfluidic production of monodisperse and multifunctional drug carriers with highly tunable physical and chemical properties.

Moreover, the specificity of cellular targeting of niosomal drug delivery systems can be further improved by active targeting for tumor therapy, by using a ligand coupled to the surface of niosomes that can be actively taken up, for example, via receptor-mediated endocytosis [28]. The addition of polyethylene glycol (PEG) to a niosome construction provides protection against the immune response and allows for further surface modifications. Tumor homing peptides, with specificity for tumors and membrane penetration, facilitate the targeted accumulation of drugs into tumor tissues and offer a solution to the poor penetration of chemotherapeutics into tumors [29]. By coupling a tumor homing peptide to the niosome surface, a penetration of tumor cells can be achieved, which further improves the efficiency of the drug delivery via active targeting [30,31]. CGNKRTR (tLyp-1) is one of the most promising homing peptides, which penetrates tumor cells through a neuropilin-1 (NRP-1) mediated endocytosis via the C-end Rule (CendR) internalization pathway. NRP-1 is overexpressed on the surface of both glioma and endothelial cells of angiogenic blood vessels [32].

Herein, we report a facile synthesis of TPT-containing niosomes as a novel drug delivery system via a microfluidic synthesis. We optimized the formulation and flow rates of the TPT-loaded polyethylene glycolated niosomes (PEGNIO/TPT) to achieve drug-loaded vehicles with a very narrow particle size distribution. A subsequent conjugation with the tumor-homing peptide tLyp-1 allowed a targeted glioblastoma therapy. The effect of different niosomal formulations on human glioblastoma cells was investigated. The obtained results are promising for the establishment of a reproducible and high-throughput fabrication method and prove an improved efficiency of the drug delivery system.

## 2. Results and Discussion

### 2.1. Optimization and Characterization of Niosomal Formulations

Recent studies suggest that organic nanoparticles such as liposomes can be rapidly synthesized in a reproducible manner using the microfluidic NanoAssemblr^TM^ system (Precision Nano-Systems Inc., Vancouver, Canada) [25,33,34]. The formation of small unilamellar nanoparticles occurs due to a steadily increasing polarity during the flow through the chamber. The effect of aqueous media on the physicochemical characteristics of the niosomes prepared by microfluidics, as well as their stability under different conditions and their in vitro cytotoxicity were tested by Obeid et al., and were compared with the ones prepared through the conventional thin-film hydration and heating block methods [20]. According to the results, by microfluidic mixing, monodisperse particles could be prepared easily in a single step with high reproducibility, and no adverse effects on particle stability and toxicity were observed [20] The flow rate ratio of solvent/aqueous phase was another parameter that strongly affected the particle characteristics, and must be optimized in order to produce vesicles with a defined size, which was important in developing an effective drug delivery system [25].

**Figure 1:**
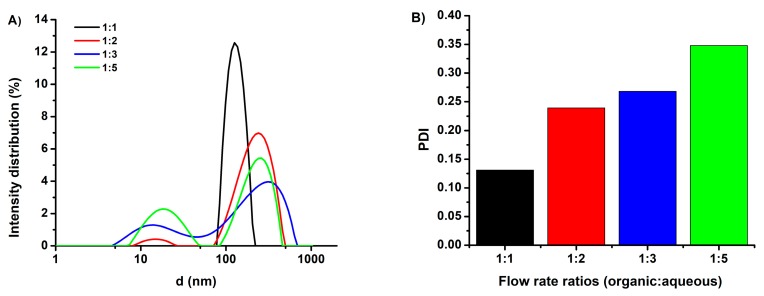
Hydrodynamic diameter (**A**) and PDI (**B**) of the topotecan (TPT)-loaded niosomes prepared using different organic: aqueous flow rate ratios (1:1; 1:2; 1:3; 1:5).

Since the microfluidic preparation of niosomes was a promising method for a variety of formulations, we investigated the influence of the flow rate ratio of the organic phase to the aqueous stream, which contained the therapeutic agent topotecan. The flow rate ratio of the organic/aqueous phases was varied from 1:1 to 1:5, and the influence of this parameter on the resulting particle size and size distribution was studied. After synthesis and subsequent purification, the mean diameters of TPT-loaded niosomes were measured by a Litesizer^TM^ 500 device and are shown in Figure 1 along with the corresponding polydispersity index (PDI) values. It can be seen from Figure 1A, that as the organic/aqueous flow rate ratio was decreased, bimodal size distributions with two maxima (first one approximately 13–18 nm, second one approximately 240–320 nm) were obtained. The formation of the small particles (13–18 nm) can be attributed to a reduced final solvent concentration. Figure 1B indicates an increase of PDI values with the shift in the organic /aqueous flow rate ratio. In contrast, the TPT-loaded niosomes formed at the 1:1 flow rate ratio showed a monodisperse particle size distribution with a mean size of 128.5 nm and a PDI of 0.13. Similarly, Kastner et al. prepared liposomes via NanoAssemblr and obtained low PDIs at a low flow rate ratio (1:1) in comparison to a higher flow rate ratio (1:5) [25]. According to these results, an organic/aqueous flow rate ratio of 1:1, which provides monodisperse particles, was utilized for all further investigations. The physicochemical properties of drug carriers, as well as their size, shape, and surface chemistry, played a critical role in determining tissue penetration, cellular delivery, and therapeutic efficacy [35]. Therefore, after the optimization of the flow rate ratio, the mean diameters of TPT-loaded niosomal formulations, TPT entrapment efficiency (EE%), and zeta potential values were investigated, and the results are listed in Table 1. For plain niosomes that were synthesized at the 1:1 flow rate ratio, a size of 138.9 nm was measured, with a PDI of only about 0.07 implying a narrow particle size distribution. Hence, our results indicated that the microfluidic preparation of plain niosomes provided lower PDI values in comparison to the thin film hydration method, which had been used in our previous studies [28,30]. The morphology of the niosomes was characterized by TEM, utilizing a negative staining procedure using a 2% aqueous phosphotungstic acid reagent. Figure S1 shows a representative TEM photomicrograph, indicating that the niosomes possess spherical shapes that could be preserved during sample preparation. The zeta potential of the bare niosomes was measured as −27.80 mV. TPT loading did not significantly influence the zeta potential of the plain vesicles (−27.00 mV) as a result of any TPT intercalation in the vesicle membrane [7]. Conjugation of tLyp-1 increased the hydrodynamic diameter of niosome, corresponding to the presence of the peptide on the niosomal surface. Surface functionalization with tLyp-1 lowered the zeta potential to −20.2 mV. The observed decrease in the absolute value of the zeta potential of the niosomes after functionalization can be explained by the presence of the positively charged peptide on the nanoparticle surface [36]. The encapsulation efficacy (EE%) of TPT was calculated to be 39.3 % for niosomes and 37.5 % after a subsequent functionalization with tLyp-1. The stability of PEGNIO/TPT/tLyp-1 was tested via DLS analysis and no changes were observed in the size and PDI values after one-month storage at 4 °C in the dark (data not shown). Furthermore, PEGNIO/TPT/tLyp-1 was diluted in cell culture media and phosphate buffered saline (PBS) and was incubated at 37 °C for 24 h. After incubation, the particle size of the samples was measured, and no changes were observed.

### 2.2. Drug Release

**Figure 2:**
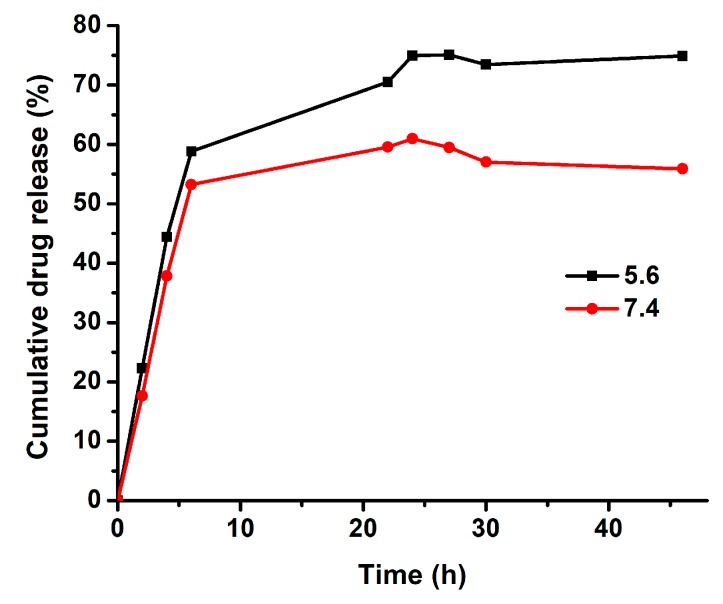
In vitro cumulative release of TPT from polyethylene glycolated niosomes (PEGNIO)/TPT/tLyp-1 at pH 7.4 and 5.6.

Topotecan is an S-phase-specific drug and needs a prolonged release profile to efficiently act against tumor cells [37]. Nanoscale drug delivery systems can offer such a sustained release of TPT. The main mechanisms determining the release rate of the drug were desorption of the drug, drug diffusion from the nanoparticle matrix, matrix erosion, and degradation of the nanoparticles [38]. The release profile of TPT from nano-formulations was evaluated in different studies [5,39,40]. Vali et al. prepared TPT-loaded PEGylated and conventional liposomes and investigated the release profiles of TPT from liposomes in PBS at pH 7.4 and in human plasma. In both liposomal formulations, the release profiles showed a prolonged release of up to 48 h [39]. Here, the release profile of TPT from niosomal drug carrier system was investigated via a dialysis method, which was one of the most common methods for the determination of drug release from nanoparticles. The analysis was carried out in simulated conditions of normal human tissue (pH 7.4) as well as in an acidic environment (pH 5.6) similar to the tumor. Samples were taken at specific intervals and measured by fluorescence emission measurements at 530 nm to determine the released amount of TPT. TPT release profiles from PEGNIO/TPT/tLyp-1 showed a faster release of TPT under acidic environments than that at neutral pH (Figure 2). This can be explained by increasing solubility of TPT with decreasing pH as a result of protonation [7,41]. Moreover, the quantity of released TPT was significantly enhanced at pH 5.6, and there seemed to be a saturation effect under neutral conditions. Within 30 h, the release of TPT amounted to 57% and 73% at pH 7.4 and pH 5.6, respectively. The release of free TPT through the dialysis membrane has been investigated already by Souza et al. and was reported to reach approximately 75% within 4 h [5]. With the designed niosomal drug delivery system, the drug release was prolonged to 30 h by passive transport of the drug through the membrane bi-layer. Hence, our results proved that PEGNIO/TPT/tLyp-1 allowed to control and extend the release of the encapsulated TPT, rendering it a promising drug delivery system.

### 2.3. Cellular Uptake and Internalization

The design of novel targeted drug delivery systems is a promising approach to achieve better clinical outcomes in cancer treatment while avoiding side effects due to high drug dosage. Overexpressed receptors on cancer tissues are suitable targets for a more specific delivery. Peptides as targeting ligands possess a number of advantages such as their accessibility of high-throughput screening, ease of synthesis, high specificity, and affinity [42,43]. Recent studies showed that tLyp-1 peptide-conjugated nanoparticles selectively home to tumor tissue, penetrate the blood vessels, and enter the tumor parenchyma [44,45]. Wu et al. conjugated the tLyp-1 peptide to fluorescent and radio-label markers that selectively bound to U87 cells at low concentrations in vitro and accumulated in NRP-positive tumors in vivo [44]. Moreover, tLyp-1 has been conjugated to niosomes for the study of targeted co-drug delivery. The specificity of tLyp-1 targeted niosomes was investigated on hMSC and U87 cells, with a more specific uptake by U87 cells compared to hMSC cells being reported [30].

**Figure 3:**
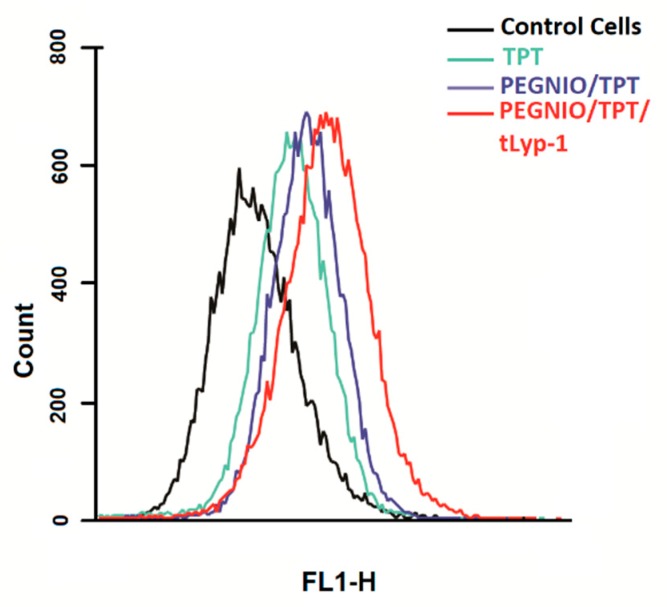
Flow cytometric measurement of TPT uptake by U87 cells after incubating with free TPT, PEGNIO/TPT, and PEGNIO/TPT/tLyp-1.

**Figure 4:**
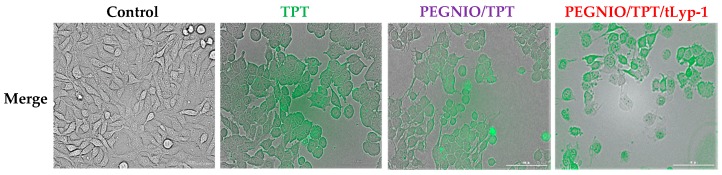
Fluorescence microscopy images of U87 cells without treatment and after incubation with TPT, PEGNIO/TPT, and PEGNIO/TPT/tLyp-1. The obtained fluorescence images were merged into the bright field picture (merge).

The ability of tLyp-1 conjugated niosomes to deliver TPT intracellularly was of significant importance and provides for a high specificity in delivery to NRP-1 receptor-overexpressing cancer cells due to the pH-sensitive structure of topotecan. Here, flow cytometry was used to investigate the total TPT uptake by U87 cells for different TPT formulations. The cells were treated with samples for 2 h. Untreated control cells and treated cells were analyzed using a BD Accuri C6 flow cytometer. As shown in Figure 3, the cellular TPT level of PEGNIO/TPT/tLyp-1 in U87 cells was significantly higher than that of PEGNIO/TPT and free TPT (note that the number of cells on the abscissa is displayed in logarithmic scale). A similar improvement of cellular uptake of the drug by the encapsulation of TPT into the vesicles and the further use of targeting ligands on the vesicle surface has been reported previously [37]. Free TPT enters the cells by diffusion [41], whereas PEGNIO/TPT and PEGNIO/TPT/tLyp-1 enter the glioma cells through endocytosis and receptor-mediated endocytosis via the NRP-1 receptor, respectively [46]. A Cytation 5 device was used to monitor the cellular internalization of TPT, PEGNIO/TPT and of the targeted niosomal TPT formulation. It can be deduced from the results that PEGNIO/TPT/tLyp-1 effectively bound to U87 cells, resulting in a high fluorescence signal of the cell, thereby demonstrating successful internalization (Figure 4). In contrast, fluorescence was significantly lower when only TPT was applied to the cell culture, indicating less uptake in U87 cells. Fluorescence microscopy images thus show results similar to the flow cytometry analysis.

### 2.4. Cytotoxicity

**Figure 5:**
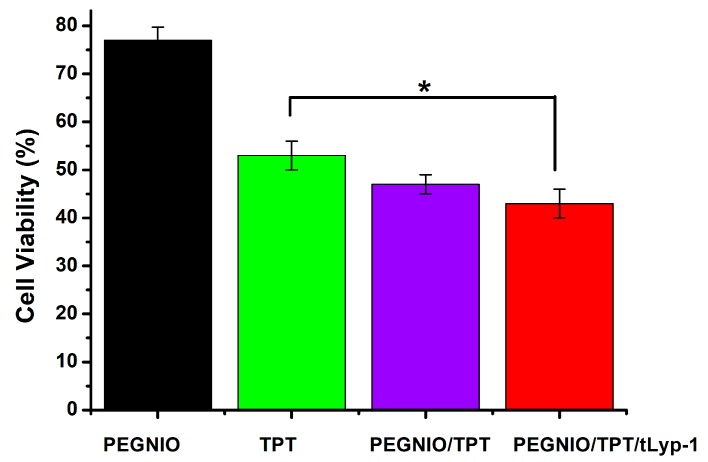
Cytotoxicity of the free drug TPT, as well as of different niosomal formulations on U87 cells. The error bars represent the standard deviation from the mean value (*n* = 3). * *p* <0.05, indicating statistically significant results.

TPT is well-established for the treatment of several cancer types, including glioma, small-cell lung, and ovarian cancers [47,48]. TPT acts as a DNA topoisomerase 1 inhibitor, thus leading to DNA cleavage and cell death (apoptosis). Here, a CellTiter-Blue (CTB) assay was used to determine the cytotoxicity of free TPT, TPT-loaded formulations, and bare niosomes on U87 cells. The cells were incubated with PEGNIO, PEGNIO/TPT, PEGNIO/TPT/tLyp-1, and free TPT (at the equivalent concentration of loaded TPT) for 24 h. The obtained values of cell viability are presented in Figure 5. PEGNIO showed to be practically nontoxic to U87 cells with relative cell viabilities of around 80% [28]. PEGNIO/TPT was slightly more toxic than free TPT, which is attributed to the fact that PEGNIO/TPT was taken up by the cells more efficiently (Figure 3). Drummond et al. have shown previously that liposomal TPT improves cellular uptake under physiological conditions and presents increased cytotoxic effects in comparison to free TPT [37]. Additionally, TPT is unstable in physiological conditions and physiological pH strongly triggers reversible hydrolysis from a closed lactone ring to the inactive carboxylated form [49], causing full loss of the antitumor activity of the drug. Therefore, here, the improved TPT cytotoxicity against U87 cells, observed using niosomes, can be attributed to both an increase in cellular uptake of TPT and protection of the TPT lactone form. Due to the conjugation of the targeting ligand to PEGNIO/TPT, PEGNIO/TPT/tLyp-1 showed significantly stronger toxic effects on U87 cells compared to free TPT after incubation for 24 h (*p* < 0.05, Figure 5). This difference in cytotoxicity was mainly resulting from the targeting ability of PEGNIO/TPT/tLyp-1 to the U87 cells expressing the NRP-1 receptor [32,50]. The tLyp-1 peptide actively binds to the overexpressed NRP-1 on glioma cells, leading to the rapid internalization of the nanocarriers through receptor-mediated endocytosis.

## 3. Materials and Methods

### 3.1. Materials

DSPE-PEG(2000) maleimide was provided by Avanti (Alabaster, AL, USA). Sorbitan monostearate (Span60), cholesterol, Dulbecco’s Modified Eagle Medium (DMEM) was obtained from Sigma Aldrich (Munich, Germany). CellTiter-Blue viability assay kits were purchased from Promega Corporation (Madison, WI, USA). Topotecan was purchased from Cayman Chemical (Ann Arbor, MI, USA). tLyP-1 peptide (CGNKRTR) was ordered from GeneCust (Ellange, Luxembourg).

### 3.2. Preparation and Optimization of Tpt Encapsulated Niosomes by Microfluidics

PEGylated niosomes and TPT-loaded niosomes were prepared using a method based on microfluidic mixing. The microfluidic instrument NanoAssemblr^TM^ (Precision Nano-Systems Inc., Vancouver, Canada), which enabled the controlled formation of vesicular structures via nanoprecipitation, was used [51]. Span 60, cholesterol, and DSPE-PEG(2000) maleimide were dissolved in 1.0 mL chloroform with a specific molar ratio (9.9: 9.9: 0.2). The resulting stock solution was used as a lipid phase and was utilized together with the acidic aqueous solution of TPT (0.02 mM) to obtain TPT-loaded niosomes. The lipid phase was injected into one inlet and the aqueous phase into the other inlet of the microfluidic micromixer, whereas the heating block was set at 65 °C Different flow rates of the lipid phase to aqueous phase (1:1, 1:2, 1:3, 1:5) were applied with the same total flow rate of 12 mL/min. The final dispersions were collected from the outlet stream and immediately dialyzed against water for 4 h.

### 3.3. Conjugation of TLyp-1 to PEGNIO/TPT

**Figure 6:**
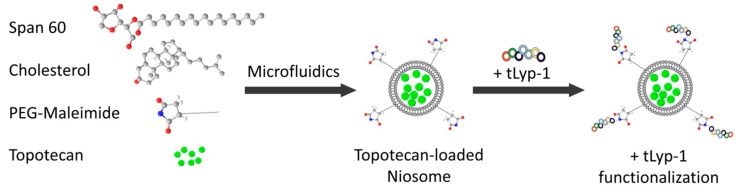
Schematic representation of the niosome preparation and the bioconjugation processes.

tLyp-1 targeted niosomes were prepared via the formation of a thioether linkage between the thiol group of tLyp-1 and the maleimide terminal group of the PEG chains on the niosomes [30]. The tLyp-1 peptide was dissolved in 50 mM HEPES buffer at pH 6.5 at a concentration of 200 µg/mL. Overnight at room temperature, 50 µL of peptide solution was reacted with the niosome dispersion (950 µL). The products were then purified using a 14 kDa dialysis bag to remove the unconjugated peptides. A schematic representation of the fabrication process consisting of the niosome preparation, drug encapsulation, and the bioconjugation steps is shown in Figure 6.

### 3.4. Measurement of Particle Size, Distribution, and Zeta Potential

The particle size distribution and zeta potential of the niosomal formulations were determined by a LitesizerTM 500 device (Anton Paar GmbH, Graz, Austria). The polydispersity index (PDI) was given as a measure of the width of the size distribution. All measurements were performed 3 times at room temperature. The morphology of the niosomes was examined using a transmission electron microscopy (TEM) (FEI Tecnai G2 F20 TMP-TEM instrument, Hillsboro, OR, USA). Briefly, a niosome sample was applied onto a carbon-coated copper grid. The remaining liquid was removed by blotting onto filter paper. Then, staining with 2% aqueous phosphotungstic acid was performed without removing the excess liquid but allowing evaporation. The samples were observed under the TEM device microscope at an accelerating voltage of 120 keV in a bright-field image mode.

### 3.5. Stability

The stability of niosomal formulations was tested via dynamic light scattering (DLS) analysis. Samples were stored at 4 °C in the dark. The particle size and PDI values were measured repeatedly over a period of 1 month. Moreover, the particle size of PEGNIO/TPT/tLyp-1 was measured in cell culture media and PBS after incubation at 37 °C for 24 h.

### 3.6. Entrapment Efficiency

The encapsulation efficiency (EE%) was utilized to express the percentage of the drug entrapped in niosomes referred to the initial amount of drug in the aqueous phase. 50 µL of the dispersion of purified niosomes was diluted with 100 µL of methanol and the niosomes were separated by centrifugation at 130,00× *g* for 5 min. This step results in the breakage of niosomal membranes and the release of the encapsulated drug. A calibration curve was derived with a known concentration of free TPT by fluorescence emission measurements at 530 nm using a Nano-Drop 3300 device. Therefore, stock solutions of TPT were prepared at 1.0 mg mL^-1^ in methanol and further diluted with methanol to the concentration range of 0.1 to 5 µg/mL. The amount of TPT in purified and non-purified samples was calculated according to the calibration curve (y = 1443.3x + 49.589, R^2^ = 0.9996) via measuring the fluorescence emission intensity at 530 nm.

### 3.7. Drug Release

In vitro drug release experiments were performed via the dialysis technique. PEGNIO/TPT/tLyp-1 was prepared and transferred into a pre-washed dialysis tubing (Slide-A-Lyzer MINI Dialysis Devices, 10K MWCO, Thermo Fisher Scientific Inc., Waltham, MA, USA). The tubing was immersed in 10 mL of the phosphate buffered saline (PBS) buffer (with a pH of 5.6 or 7.4), placed in an incubator at 37 °C, and stirred at 100× *g*. At specific time intervals, samples of 0.5 mL were removed from the release medium and replaced with the same volume of fresh buffer. The amount of released TPT was calculated according to the calibration curves.

### 3.8. Cell Culture

U87 (glioblastoma cells) cell lines were provided by the German Collection of Microorganisms and Cell Cultures (DSMZ). U87 cells were grown in DMEM containing 10% fetal calf serum (FCS) (Biochrom GmbH, Berlin, Germany) and 1.0% penicillin/streptomycin (P/S).

### 3.9. Cellular uptake and internalization

The uptake of the TPT-loaded niosomal formulations and free TPT by U87 cells was analyzed by flow cytometry. The cells (5 × 10^5^) were treated with free TPT, PEGNIO/TPT, and PEGNIO/TPT/tLyp-1 for 2 h, and treated cells were washed 2 times with PBS and then analyzed in a BD Accuri C6 cytometer. Cellular internalization of samples was determined via fluorescence microscopy studies. U87 cells were cultivated for 2 days on the 96 well plates in a volume of 200 µL of the medium. Samples were diluted with medium and then added to the cells. The cells were incubated for 4 h at 37 °C and washed once in PBS. Images were taken using a Cytation 5 imaging reader (BioTek Instruments, Inc., Winooski, VT, USA).

### 3.10. Cytotoxicity

The metabolic activity of viable cells in terms of their reduction capacity of resazurin was measured via a CTB assay (Promega Corp., Madison, WI, USA) [52]. The cytotoxic effects of niosomal formulations and free TPT were tested on U87 cells. Cells (8 × 10^3^) were seeded out in 96-well tissue plates (Sarstedt, Nümbrecht, Germany) in a volume of 200 µL and cultivated for 3 days. After this cultivation time, cells were washed once with PBS and treated with free TPT, PEGNIO/TPT, PEGNIO/TPT/tLyp-1 for 24 h. The equivalent concentration of free TPT was used in niosomal formulations. Then the samples were removed and 100 µL of CTB reagent (diluted 1: 6 with supplement-free DMEM medium) was added to each well and incubated for 1 h (37 °C, 5% CO_2_). The resulting fluorescence intensities (544Ex/590Em) were recorded with a fluorescence spectrometer Fluoroskan Ascent (Thermo Fisher Scientific Inc., Waltham, MA, USA).

### 3.11. Statistical Analysis

Statistical data analysis was performed using the Student’s t-test. The difference between the 2 groups was considered to be significant when the *p*-value was less than 0.05.

## 4. Conclusions

In this work, the rapid microfluidic preparation of a targeted niosomal drug delivery system was developed to improve the therapeutic efficacy of TPT for glioma. Microfluidic mixing ensures the preparation of niosomes with controlled size and the parallel loading of topotecan. A simple strategy for the TPT encapsulation procedure into niosomes was established, and the obtained formulations were characterized in detail. The flow rate ratio was identified as a key process parameter and varied to produce niosomes of a defined size, which is quite important in developing an effective drug delivery system. Thus, TPT-loaded niosomes with a mean size of about 130 nm and with a very narrow size distribution could be obtained via microfluidics. Subsequently, the surface of the drug-loaded niosomes was decorated with tLyp-1 as tumor homing and penetrating peptide. The targeted drug-loaded nanoparticles show substantially stronger cytotoxicity owing to binding to the NRP-1 receptor-overexpressed U87 cells. Our results will be highly useful as a first step towards a rational optimization of niosome manufacturing for drug delivery approaches.

Due to the continuous microfluidic synthesis that can be scaled up by parallelization and showed to be robust for the formation of relatively uniform niosomes, as well as all subsequent functionalization steps that can be performed in a quantitative fashion, and show good potential to be carried out at larger scales. This niosomal system offers new opportunities for the development of novel nanomedical products at industrial scales.

## Figures and Tables

**Table 1:** Characterization of niosomal formulations.

Samples	EE (%)	Size (nm)	PDI	Zeta Potential (mV)
PEGNIO	−	138.90	0.072	−27.80
PEGNIO/TPT	39.30	128.47	0.131	−27.00
PEGNIO/TPT/tLyp-1	37.50	159.79	0.124	−20.20

Abbreviations: PDI: Polydispersity index; EE: Encapsulation efficiency %.

## Supplementary Materials

The following are available online at https://www.mdpi.com/1422-0067/20/19/4696/s1, Figure S1: TEM micrograph of niosomes stained with 2% phosphotungstic acid.
