# Supplementary material for: Rapid Microfluidic Preparation of Niosomes for Targeted Drug Delivery
Source: Int J Mol Sci. 2019 Sep 22;20(19):4696. doi: 10.3390/ijms20194696 (PMC6801367; doi:10.3390/ijms20194696)
Supplement: Supplementary file 1 [file ijms-20-04696-s001.pdf]

Rapid microfluidic preparation of niosomes for targeted drug delivery

Didem Ag Seleci, Viktor Maurer, Frank Stahl, Thomas Scheper and Georg Garnweitner

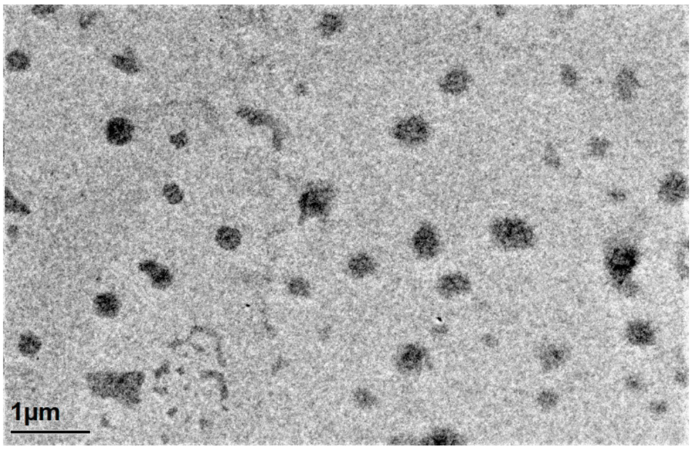

Figure S1. TEM micrograph of niosomes stained with 2 % phosphotungstic acid.
